# Perceptions of Environmental Influence and Environmental Information-Seeking Behavior Among People With Asthma and COPD

**DOI:** 10.3389/fdgth.2022.748400

**Published:** 2022-05-03

**Authors:** Shivani Parikh, Kelly Henderson, Rahul Gondalia, Leanne Kaye, Esther Remmelink, Alesha Thompson, Meredith Barrett

**Affiliations:** ^1^Harvard T. H. Chan School of Public Health, Environmental Health, Boston, MA, United States; ^2^ResMed Inc., Science Center, San Diego, CA, United States; ^3^Propeller Health, User Research, San Francisco, CA, United States; ^4^Propeller Health, Data Analytics, San Francisco, CA, United States; ^5^Council of State and Territorial Epidemiologists, Programs, Atlanta, GA, United States

**Keywords:** environmental exposures, asthma, COPD, SES, digital health, self-management, smartphone app, health information

## Abstract

Environmental exposures and socioeconomic status (SES) are associated with asthma and chronic obstructive pulmonary disease (COPD) morbidity and mortality. Despite efforts to reduce the impact of environmental exposures through regulation and education, knowledge gaps remain. We sought to understand how adults with asthma and COPD perceive and seek information about environmental factors, and how these responses varied by disease or socioeconomic characteristics. Participants with self-reported asthma or COPD enrolled in a digital platform for respiratory disease self-management, consisting of sensors to track medication use and a companion smartphone app, completed an electronic survey exploring perceptions of environmental factors. Using mixed-method analyses, we evaluated differences in responses by disease (asthma vs. COPD), education (≤ vs. > some college), annual household income (< vs. ≥ $50,000), and mean annual residential air pollutant exposure (> vs. ≤80th percentile). Survey responses from 698 participants [500 asthma (72%) and 198 COPD (28%)] were analyzed. A high percentage of participants perceived that environmental factors could influence their symptoms, including: pollen (93% for asthma vs. 86% for COPD), mold (89 vs. 85%), second-hand smoke (89 vs. 83%), and air pollution (84% for both). Participants reported seeking environmental information daily from an average of three sources, preferring mobile apps and television (TV) programs. Significant differences were identified by disease.

**Conclusion:**

Participants with asthma and COPD perceive a relationship between their respiratory symptoms and their environment and regularly seek out environmental information. This information can help inform digital health development for respiratory education and self-management.

## Introduction

Chronic respiratory diseases, including asthma and chronic obstructive pulmonary disease (COPD), are among the leading causes of morbidity and mortality worldwide (1). In the US, asthma affects 25 million people (2), while COPD affects 16 million people (3); both lead to significant social burden with over 30 million days of missed work and school estimated (4, 5). Environmental factors such as air pollution have been associated with respiratory mortality (6, 7) and worsening of symptoms including cough, dyspnea, and wheeze (8).

These respiratory disease burdens disproportionately affect minority and socioeconomically-disadvantaged populations, who are more likely to be exposed to poorer air quality and experience higher respiratory prevalence and morbidity (9). Asthma prevalence is higher in adults with household incomes 100% below the federal poverty level (FPL) (10.8%) than in those with incomes >450% of the FPL (6.5%) (10), and mortality is negatively associated with both education and income (11). Similarly, COPD prevalence is higher in adults with household incomes below the FPL (8.3%) than in those with incomes >200% of the FPL (4.3%), and disease severity is negatively associated with socioeconomic status (SES) (12).

Education about environmental factors is recommended by national clinical guidelines as a component of regular asthma management (13). Education may improve self-management among chronic respiratory disease patients of all SES levels by reducing health literacy barriers, which could lead to reductions in symptoms, healthcare utilization, and absenteeism (9, 14). Early evidence suggests digital health may serve a role in providing education to people with respiratory disease (15); however, further research is needed to design content delivery in usable and convenient ways that can serve users from different socioeconomic strata with varying levels of health literacy (16).

In order to better understand perceptions of environmental factors among participants with asthma and COPD, this study aimed to evaluate: (1) preferred sources for information about environmental factors; (2) knowledge and perceived impact of environmental exposures on symptoms; and (3) perceived agency to limit environmental impact on symptoms. Lastly, we aimed to assess how these perceptions varied by disease, education, income and mean air pollutant exposure.

## Methods

### Participant Recruitment and Enrollment

Existing users of a digital health platform for respiratory management (Propeller Health, CA, USA) were invited to participate in an online survey through an email invitation. Participants self-reporting either asthma or COPD, ≥18 years, residing in the US, and who self-enrolled online between December 2017 and June 2019, largely within Facebook campaigns, were eligible. Participants who had at least one sensor data transmission (“sync”) in their first 30 days on the platform were included. Eligible participants provided electronic consent before taking the survey, and those who completed the survey were entered into a raffle for a $150 Amazon gift card. The study protocol was approved by the Copernicus Independent Review Board (Protocol # 20191728).

### The Digital Health Platform

The digital platform, consisting of a small electronic medication monitor (EMM) and an accompanying smartphone application (“app”), passively collects inhaler use data from users with asthma and/or COPD. The EMM attaches to a participant's inhaler medication, captures the date and time of each inhaler use, and provides objective information about adherence to controller medications and use of rescue medications. The EMM then relays these data (“syncs”) to a paired smartphone, which captures the geographic location of use. In the platform, each rescue medication use event is also assigned local environmental exposure levels for five criteria air pollutants [fine particulate matter (particles <2.5 μm in diameter; PM_2.5_), coarse particulate matter (particles <10 μm in diameter; PM_10_), ozone (O_3_), nitrogen dioxide (NO_2_), and sulfur dioxide (SO_2_)] and weather conditions (e.g., temperature, precipitation, pressure, and visibility). These data are then used to create personalized reminders, feedback and education in the app.

**Figure 1:**
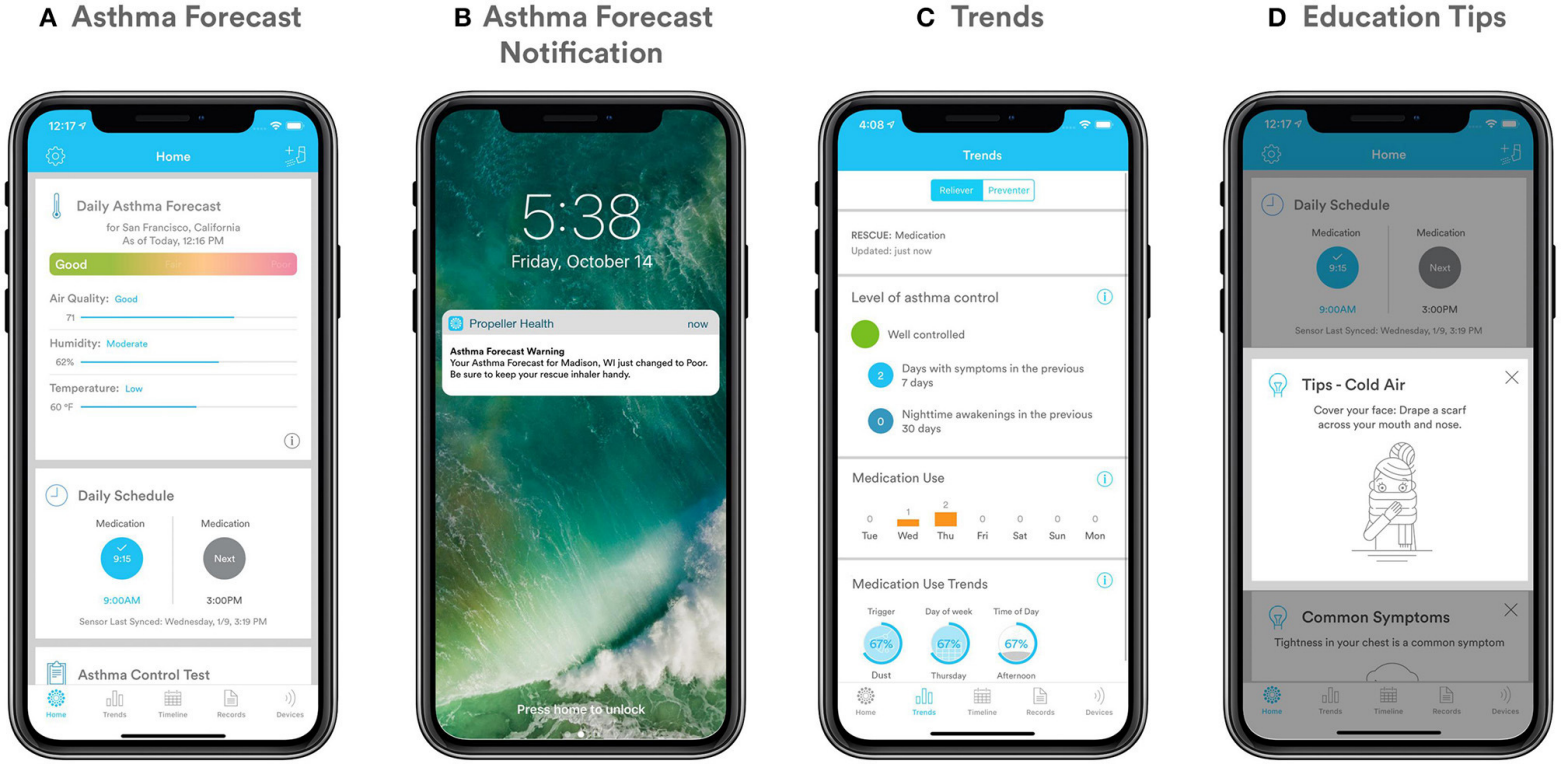
**(A–D)** Personalized components of the app **(A)** Asthma Forecast; **(B)** Asthma Forecast notification; **(C)** Trigger tracking and trends; **(D)** Education cards.

Three specific components of the app communicate information about environmental conditions that are relevant to asthma and COPD (Figure 1). For users with asthma, the Asthma Forecast utilizes an individual's past record of rescue medication use to predict whether they may use rescue medication on a specific day as a function of the current environmental conditions in their location (Figure 1A). Users with asthma may also receive push notifications when the Asthma Forecast transitions to “poor” or “good” in their area (Figure 1B). Trigger tracking and trends are based upon users' self-reported triggers that they perceive may be associated with their symptoms (Figure 1C). A subset of in-app education cards present content on environmental conditions relating to asthma or COPD (Figure 1D).

### Measures and Outcomes

Participants self-reported their demographic information (gender, race, age) and components of their SES (highest education level achieved and annual household income). Annual mean air pollutant concentrations including PM_2.5_, PM_10_, O_3_, NO_2_, and SO_2_ were assigned to each participant based on residential zip code using data downloaded from the U.S. Environmental Protection Agency's (US EPA) Air Quality System for 2018. Controller medication adherence (number of puffs taken divided by the number of puffs prescribed per day, capped at 100%) and total rescue medication use (puffs per person per day) were assessed using data collected by the EMM.

Self-reported questionnaires for asthma [Asthma Control Test (ACT)] (17) and COPD [COPD Assessment Test (CAT)] (18) were administered monthly through the Propeller app. An ACT score of <20 indicates uncontrolled asthma and a CAT score of >20 indicates greater COPD burden.

A 25-question asthma survey and a 20-question COPD survey were developed to assess preferred sources for information about environmental factors, knowledge and perceptions of environmental factors, and sense of agency in mitigating the impact of these factors (see full surveys in Supplemental Material 1). Answer categories were chosen based on the literature. Participants were able to select multiple answer categories for certain questions and were able to write in an unlisted option, but combinations of responses and open-ended responses were not assessed herein. The survey was delivered *via* email and data were collected *via* a HIPAA-compliant platform (Survey Monkey).

### Statistical Analyses

Survey results were analyzed for participants who completed the survey using R version 4.0.2 (19). Four closed-ended questions were analyzed by calculating the frequency at which each possible response was selected and conducting statistical analyses to assess differences in responses by participant characteristics, including *t*-tests for continuous variables and chi-squared tests for categorical variables. Logistic regressions were used to estimate the relationship between disease (COPD vs. asthma) and each study outcome. All models adjusted for age, gender, app use at 180 days (a proxy for app engagement), education, and income. Analyses comparing COPD and asthma were restricted to participants at least 40 years of age in order to standardize across age-based differences. Models were stratified by asthma and COPD to estimate disease-specific associations between education (< vs. ≥ college degree), income (< vs. ≥$50,000), and annual mean air pollution exposure (≤ vs. >80th percentile) with each response, while adjusting for age, gender, and app use at 180 days. The threshold for statistical significance was α = 0.05.

## Results

### Population Description

The analysis included 698 participants, including 500 with asthma (72%) and 198 with COPD (28%), which represented a 13% email open rate and a 99% survey completion rate across both surveys (including all demographic questions) (Table 1). Participants were predominantly female (79.0% for asthma, 56.1% for COPD) and White (80.4% for asthma, 88.4% for COPD). A larger percentage of participants with COPD had an annual household income <$50,000 (68.2 vs. 46.4% for asthma) and less than a college degree (82.3 vs. 58.8%). Among asthma participants, 82.6% (*n* = 413) had uncontrolled asthma as defined by ACT. Among COPD participants, 33.8% (*n* = 67) had higher impact COPD as defined by CAT. Annual mean pollutant exposures are reported in Table 1 and Supplementary Table S1.

**Table 1:** Population characteristics.

**Disease (*N*)**	**Asthma (500)**	**COPD (198)**	***p-*value**
**Mean age, y (SD)**	37.8 (12.3)	60.3 (9.1)	<0.001
**Aged** **≥** **40 y**, ***n*** **(%)**	197 (39.4)	194 (98.0)	<0.001
**Female**, ***n*** **(%)**	395 (79.0)	111 (56.1)	<0.001
**White**, ***n*** **(%)**	402 (80.4)	175 (88.4)	0.02
**Education** **<** **college degree**, ***n*** **(%)**	294 (58.8)	163 (82.3)	<0.001
**Annual household income** **<** **$50,000**, ***n*** **(%)**	232 (46.4)	135 (68.2)	<0.001
**Annual mean exposure, unit (SD)**			
Mean PM_2.5_, μg/m^3^ (SD)	7.97 (1.77)	8.06 (1.76)	
Mean PM_10_, μg/m^3^ (SD)	17.36 (7.34)	17.26 (6.28)	
Mean O_3_, ppb (SD)	40.74 (4.28)	40.91 (3.86)	
Mean SO_2_, ppb (SD)	1.83 (2.49)	2.35 (4.03)	
Mean NO_2_, ppb (SD)	18.91 (8.25)	17.29 (9.05)	
**<20 ACT (uncontrolled asthma)**, ***n*** **(%)**	413 (82.6)		
**>20 CAT (higher impact COPD)**, ***n*** **(%)**		67 (33.8)	
**Mean SABA per person per day, puffs (SD)**	1.38 (2.42)	1.89 (2.53)	0.02
**Mean daily controller medication adherence, % (SD)**	54 (33)	64 (34)	0.004

### Preferred Sources for Environmental Information

**Figure 2:**
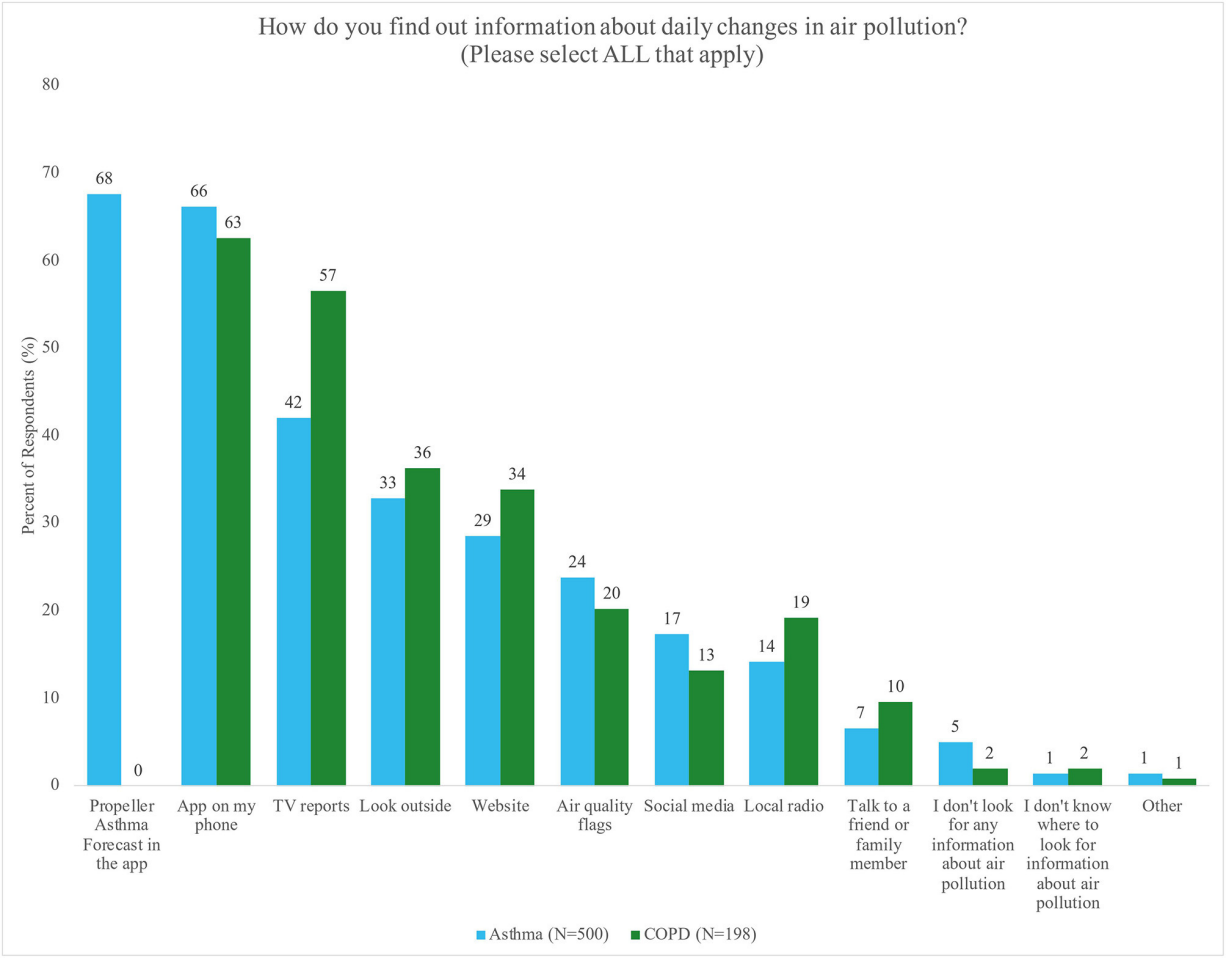
Percent of respondents with asthma (blue) or COPD (green) who reported preferred sources for seeking information about daily changes in air pollution. Participants could select more than one answer.

The survey assessed whether and how participants seek information about daily changes in air pollution (Figure 2): 95% of participants with asthma and 98% of those with COPD indicated that they sought information daily using at least one source. Participants reported an average of 3 preferred sources, with the most frequently used being the Propeller Asthma Forecast in the app (asthma only), mobile apps, and TV, in that order.

Asthma and COPD participants reported differences in preferred sources (Table 2). Participants with COPD were more likely to seek out information about air pollution than participants with asthma across multiple sources, including: mobile apps [OR = 1.89, 95% CI (1.05, 3.39), *p* = 0.03], TV reports [OR = 1.83, 95% CI (1.04, 3.21), *p* = 0.04], social media [OR = 2.28, 95% CI (0.95, 5.44), *p* = 0.06], looking outside [OR = 2.77, 95% CI (1.49, 5.17), *p* = 0.001], and talking to a friend or family member [OR = 15.00, 95% CI (2.54, 88.60), *p* = 0.003]. Furthermore, participants with COPD had lower odds of not seeking information about air pollution than participants with asthma. Significant differences by air pollution exposure and education were inconsistent, and there were no significant differences by income (Supplementary Tables S2–S6).

**Table 2:** Odds ratio (OR) of preferred sources for air pollution information as reported by participants with COPD vs. asthma.

**Response**	**OR**	**Lower 95% CI**	**Upper 95% CI**	***p-*value**
App on my phone	1.89	1.05	3.39	0.03
TV reports	1.83	1.04	3.21	0.04
Look outside	2.77	1.49	5.17	0.001
Website	1.14	0.63	2.06	0.68
Air quality flags	1.41	0.69	2.88	0.35
Social media	2.28	0.95	5.44	0.06
Local radio	1.64	0.77	3.50	0.20
Talk to a friend or family member	15.00	2.54	88.60	0.003
I don't look for any information about air pollution	0.28	0.06	1.28	0.10
Newspaper	1.36	0.48	3.86	0.56

### Knowledge and Perceptions of Environmental Influence

**Figure 3:**
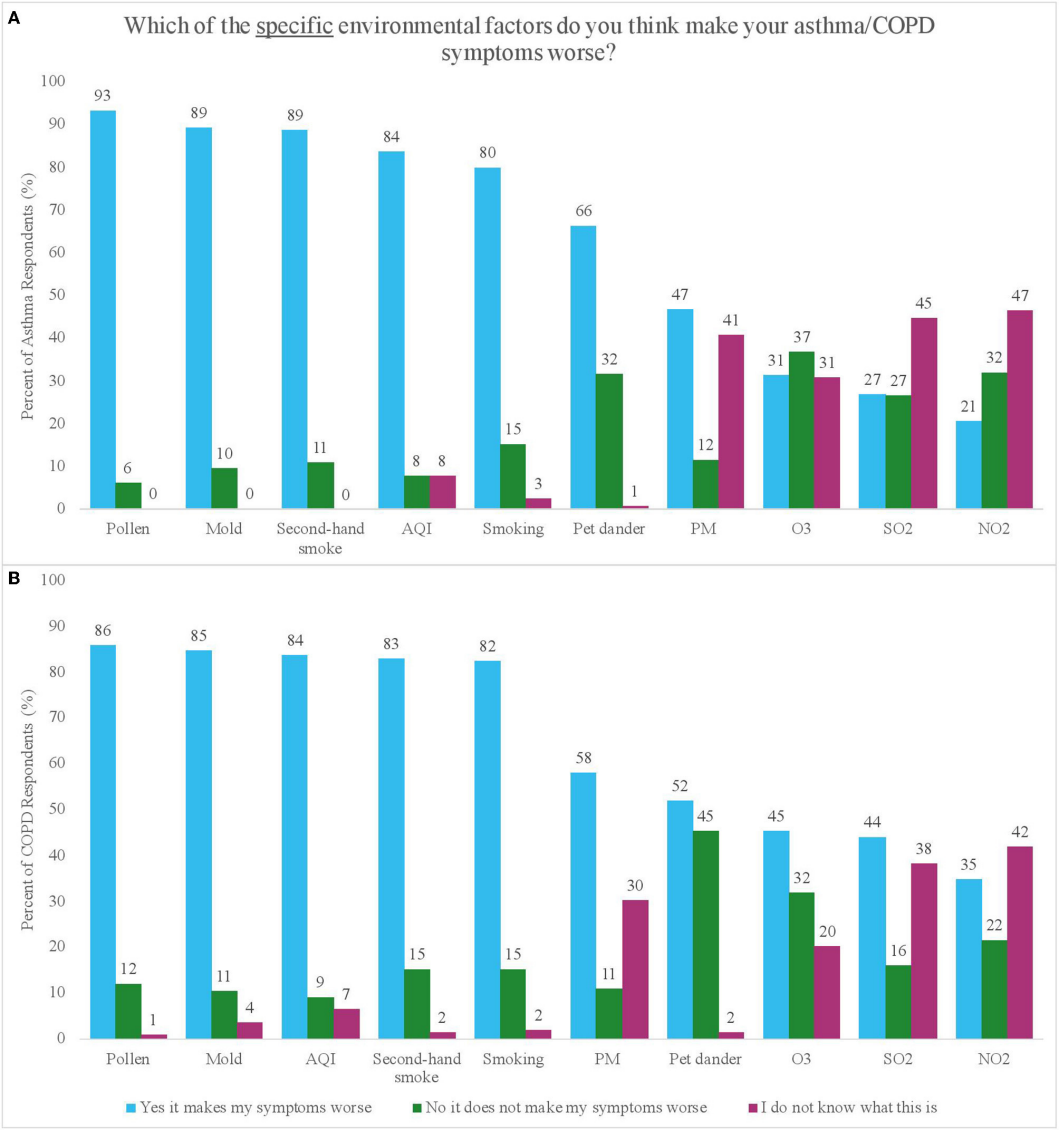
**(A,B)** Percent of respondents with asthma **(A)** and COPD **(B)** who reported perceiving or not perceiving an impact of different environmental factors on their symptoms, as well as the percentage of respondents who reported not knowing what the factor was.

The survey assessed whether or not participants perceived an influence of several environmental factors on their symptoms (Figures 3A,B). Participants perceived that pollen (93% for asthma vs. 86% for COPD), mold (89 vs. 85%), second-hand smoke (89 vs. 83%), air pollution (AQI) (84% for both), smoking (80 vs. 82%) and pet dander (62 vs. 58%) influenced their symptoms.

Among COPD participants who perceived knowing what PM was (defined as answering either “yes this makes my symptoms worse” or “no this does not make my symptoms worse” vs. “I do not know what this is”), income was significantly associated with the way they perceived PM impacting their symptoms, where the odds of believing that PM affected their symptoms were eight times higher in participants with low (vs. high) income levels [OR = 7.76, 95% CI (2.41, 24.99), *p* = 0.001] (Supplementary Table S16). There were some significant differences in whether specific air pollutants were perceived to impact symptoms by disease, education, and exposure, however these results were inconsistent (Supplementary Tables S7–S10).

We evaluated perceived knowledge about environmental factors by examining the frequency with which participants responded, “I do not know what this is,” for each environmental factor (Figures 3A,B). A small percentage of participants (asthma vs. COPD) reported not knowing what pollen (0 vs. 1%), mold (0 vs. 4%), second-hand smoke (0 vs. 2%), and AQI (8 vs. 7%) were, but a larger percentage reported not knowing what PM (41 vs. 30%), O_3_ (31 vs. 20%), SO_2_ (45 vs. 38%), and NO_2_ (47 vs. 42%) were. There were no significant differences in knowledge of specific pollutants (PM, O_3_, NO_2_, and SO_2_) by disease, education, income, or exposure (Supplementary Tables S11–S15).

### Sense of Agency to Practice Self-Management

**Figure 4:**
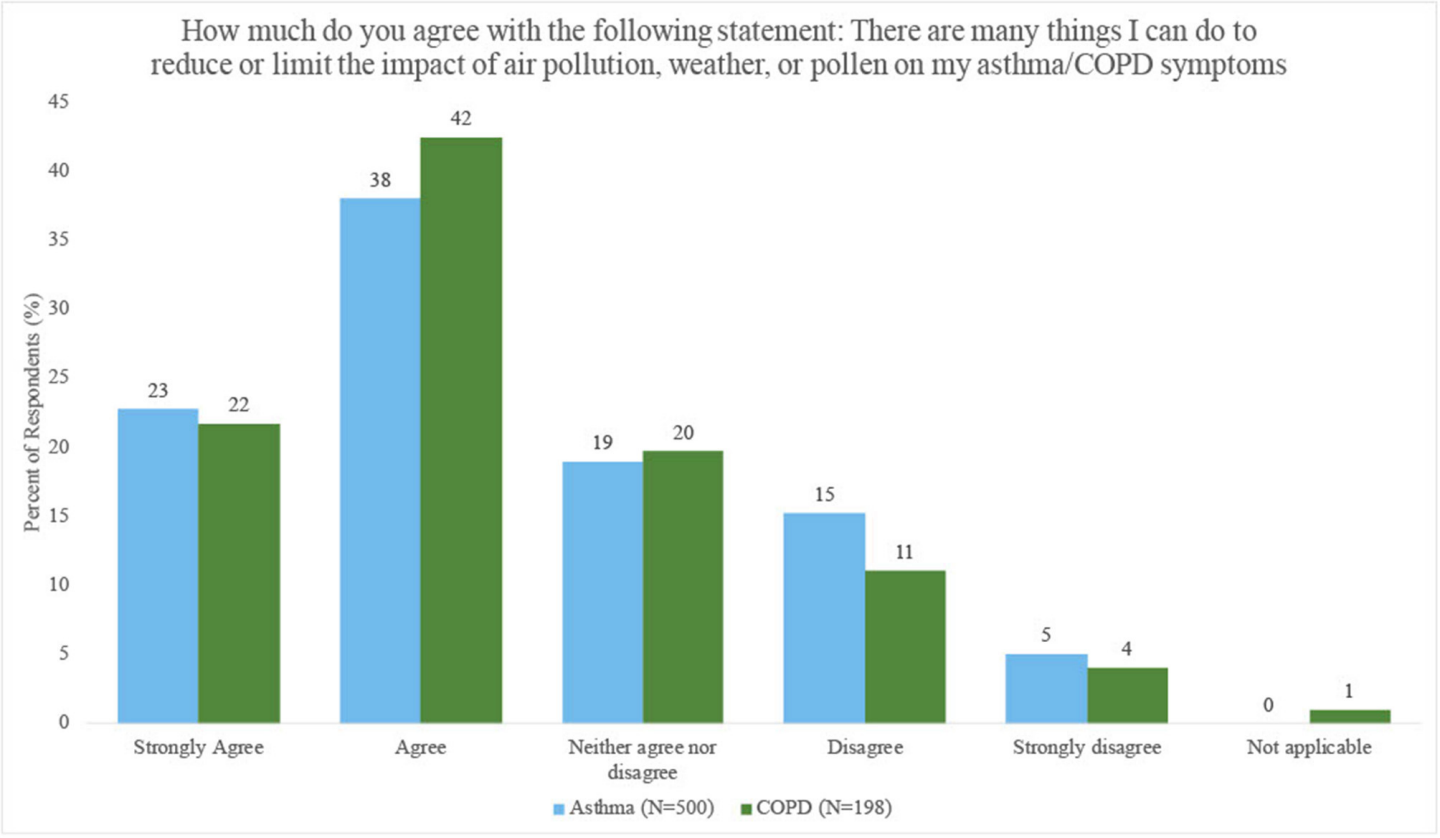
Percent of respondents with asthma and COPD who perceived they could limit or reduce the impact of air pollution, weather, or pollen on their symptoms.

The survey assessed how strongly participants felt that they could do something to “reduce or limit the impact of air pollution, weather, or pollen” on their symptoms. The majority of participants agreed (61% asthma vs. 64% COPD) that they could do something to limit the impact of environmental factors (Figure 4).

Participants exposed to high (vs. low) levels of PM_2.5_ were less likely to agree that they could limit the impact of environmental factors on their symptoms [OR = 0.61, 95% CI (0.35, 1.08), *p* = 0.09; COPD OR = 0.45, 95% CI (0.20, 1.08), *p* = 0.08], but the odds of feeling agency were higher in participants exposed to high (vs. low) levels of O_3_ [OR = 1.71, 95% CI (0.97, 3.02), *p* = 0.06; COPD OR = 2.31, 95% CI (0.80, 6.64), *p* = 0.12], however results were not statistically significant. Results were not consistent or statistically significant across other air pollutants (Supplementary Table S19). There were no significant differences by disease, education or income (Supplementary Tables S16–S18).

The survey assessed specific actions participants might take to reduce exposure and mitigate symptoms. When participants with asthma were asked, “On days when you see that the Propeller Asthma Forecast is poor, which of the following actions might you take?” Participants could select multiple answers. The top five most frequent actions included: “bring my rescue inhaler with me when I go out” (78%), “stay inside and avoid going outside as much as possible” (61%), “try to avoid my known outdoor triggers” (58%), “turn on AC/heat” (45%), and “shut windows/doors” (38%) (Supplementary Figure S1). Participants with asthma with low (vs. high) education levels were more likely to take the following actions: “bring my rescue inhaler with me when I go out” [OR = 1.53, 95% CI (0.94, 2.49), *p* = 0.08], “bring it up with my doctor” [OR = 2.81, 95% CI (0.99, 7.95), *p* = 0.05], and “talk with my family and friends” [OR = 2.39, 95% CI (0.89, 6.41), *p* = 0.08], (Supplementary Table S21). Significant differences by income and exposure were inconsistent (Supplementary Tables S22, S23).

## Discussion

This study is one of the first to evaluate perceptions of environmental impact among participants with asthma and COPD using a self-management digital health platform. Findings show that participants with asthma and COPD perceive an impact of multiple environmental factors on their respiratory disease symptoms, and they seek out environmental information daily through a variety of sources, with a preference for mobile apps and TV. Participants with COPD were more likely than those with asthma to seek information from a variety of sources. While most respondents were familiar with environmental factors such as mold, pollen and air pollution as defined by the AQI, specific air pollutants were far less familiar.

### Preferred Sources for Environmental Information

Almost all participants reported seeking information about air pollution daily. This finding is supported with Health Information-Seeking Behavior (HISB) literature, which explores the contextual factors that influence how individuals obtain information to better understand their health (20, 21). Disease severity has been associated with more active HISB (22). In this study, a majority of participants with asthma were not controlled at enrollment (82.6% ACT score <20) and one third of participants with COPD experienced high disease burden at enrollment (33.8% CAT score > 20), which may have encouraged the more active HISB seen within participants in this study. As seen in the literature, HISB can lead to improved knowledge, self-management, empowerment and symptoms (21, 22). Evaluating the impact of HISB on clinical outcomes was outside the scope of this study, but should be evaluated in future studies.

Participants with COPD were more likely to seek information about air pollution than those with asthma, and around twice as likely to rely on mobile apps, TV reports, and social media, despite older mean age. This pattern may reflect the progressive, daily impact of COPD on people's lives. Additionally, older (23) and lower-SES populations (24) tend to watch TV more frequently than their respective counterparts.

Participants sought air pollution information from an average of 3 sources. Similar findings have been reported in other populations where patients used 3–6 health information resources (25, 26) in order to validate their information (20). The use of any source will be influenced by its accessibility and credibility, which may vary for different populations (20).

Digital sources of information were popular among the majority of participants, likely driven by the convenience of on-demand, updated information. Hesse et al. (27) reported that although most patients wanted to go to physicians for health information, they actually went to the internet first, likely due to availability and convenience. In a study of 176 patients with COPD, Stellefson et al. (28) found that most used digital modalities to find health information, with no age-related differences. It was hypothesized that increased mobile device ownership may transition self-management of chronic diseases to mobile apps (28). Although these studies are part of a growing body of literature exploring the use of digital communication tools for self-management, many are limited in robustness or population representativeness, and more research is required to draw conclusions (28).

### Knowledge and Perceptions of Environmental Influence

Participants with asthma and COPD strongly perceived that environmental factors like ambient outdoor exposure, including pollen and air pollution, impacted their symptoms. Interestingly, most asthma (84%) and COPD participants (84%) were aware of air pollution (as defined by AQI) but were far less familiar with individual pollutants including PM, O_3_, NO_2_, and SO_2_. Studies have shown that increased awareness of AQI leads to greater risk perception of air pollution, which has been associated with better self-management in patients with chronic respiratory disease (29–32). For example, Wen et al. (29) reported that up to 75% of adults with asthma would change their outdoor activity patterns as a result of AQI alerts. Therefore, communicating information about AQI levels may help to mitigate exposure. However, in order to practice more targeted self-management strategies, it may be important to educate patients with asthma and COPD about specific air pollutants that may impact their health (33). For example, perceiving that O_3_ exposure negatively impacts chronic respiratory disease and knowing that O_3_ levels are highest during high-traffic, high-sunlight hours could help someone avoid the outdoors during those times (34). More focused efforts to provide education about specific air pollutants should therefore be made, but must balance detailed information with ease of presentation and usability.

Interestingly, participants perceived pollen as influencing symptoms in participants with both asthma and COPD, even though there is limited evidence documenting an allergic phenotype in COPD. Jamieson et al. (35) found that allergic status was associated with a higher risk of cough, wheeze, and exacerbations requiring treatment or an acute healthcare visit in a subset of people with COPD. However, further research is needed to define the allergic phenotype of COPD and understand its impact on COPD symptoms. Additionally, a higher percentage of participants perceived pollen and mold to influence their symptoms compared to smoking or secondhand smoke, which is unexpected given the strong evidence linking tobacco to these health outcomes compared to the limited evidence for pollen and mold. While it is possible that participants in this sample do not smoke or live in a home with smokers, we did not collect this information.

### Sense of Agency to Practice Self-Management

Most participants believed that they could limit or reduce the impact of environmental factors on their symptoms. This may be a function of knowledge of the disease or environmental factors, confidence in self-management, or participant characteristics. Improved understanding of how respiratory disease can be influenced by environmental factors may help to increase confidence in reducing their impact.

Self-management strategies like mitigation and avoidance of triggers and irritants are recommended as an effective way to improve health outcomes in asthma and COPD (34–37). In this study, the top five most frequent actions reported by participants with asthma in response to a “poor” Propeller Asthma Forecast all have been cited as examples of effective self-management strategies. Interestingly, some significant differences were found by education level, wherein asthma participants with lower education levels were more likely to bring their rescue inhaler when going out and discuss the “poor” reading with a doctor, family, and friends.

### Limitations

The findings from this study should be considered within the context of several limitations. The survey relied on self-reported data from self-selected digital health platform users and self-selected survey respondents who were possibly more engaged in their disease management and more open to digital health, thereby introducing selection bias. Differences in important factors such as education, income, race, and language between digital health users and non-users as well as survey responders and non-responders could not be assessed because of limited information availability. Lack of differences seen in this analysis across levels of education and income may be due to the homogeneity of the study population, which was overwhelmingly White and female. Additionally, low-SES populations may have faced barriers to participation in this survey since having access to email and a smartphone was required to be eligible for this study, thereby possibly introducing additional bias. Survey design and wording may have influenced the way in which respondents answered questions, and therefore findings should be confirmed with validated scales.

## Conclusions

In conclusion, this study found that participants with asthma and COPD strongly perceive a relationship between their respiratory symptoms and their environment and regularly seek out environmental information daily from apps and TV. These findings can inform digital health app development by further describing the frequency (e.g., daily vs. hourly), type (pollen, weather and air pollution), delivery (app vs. social media), visualization and level of detail (e.g., AQI vs. specific pollutant) of information that users seek to guide their self-management for respiratory disease. For instance, utilizing mobile apps to educate patients in visual, concise and compelling ways about how environmental factors influence respiratory disease may be an effective and acceptable education strategy for both people with asthma and COPD. Additionally, providing a reliable and convenient source of environmental information daily within the app, and drawing the connection to associations with respiratory symptoms, such as through the Asthma Forecast, can provide actionable information for their daily self-management and may enhance feelings of agency. Results from this study can inform the refinement of these app experiences. However, these topics are understudied and further research is needed.

## Data Availability Statement

The medication use and survey response data that supported this study are not publicly available because they are considered Protected Health Information (PHI) under the Health Insurance Portability and Accountability Act of 1996 (HIPAA) in the US, and as such are only accessible under specific authorization of access following HIPAA guidelines. The air pollution data can be accessed at the EPA Air Quality Management System (https://www.epa.gov/aqs). Requests to access the datasets should be directed to meredith.barrett@resmed.com.

## Ethics Statement

The studies involving human participants were reviewed and approved by the Copernicus Independent Review Board (Protocol # 20191728). The patients/participants provided their written informed consent to participate in this study.

## Author Contributions

KH, RG, LK, and MB conceived of the research idea and contributed to data collection. KH and MB obtained research funding. SP, RG, and ER conducted the analyses. SP, MB, and RG wrote the manuscript. All authors interpreted the results, critically reviewed the manuscript, edited, and approved the submitted version.

## Funding

This research was supported in part by Cooperative Agreement Number 5U38OT000143-05 awarded to the Council of State and Territorial Epidemiologists (CSTE) by the Centers for Disease Control and Prevention (CDC) and Climate and Health Program's Center for State, Tribal, Local, and Territorial (CSTLTS) Grant.

## Author Disclaimer

The contents of the report are solely the responsibility of the authors and do not necessarily represent the official position of the CDC.

## Conflict of Interest

KH, RG, and ER were employees of Propeller Health during the work and received salary and stock options. RG was employed by ResMed Inc. LK and MB were Propeller employees previously and are currently employees of ResMed, which acquired Propeller Health and receive salary, stock, and have pending intellectual property filings related to Propeller products. SP was a part-time employee at ResMed during the development of the manuscript. MB had full access to all data in this study and takes complete responsibility for the integrity of its analysis. AT was employed by Council of State and Territorial Epidemiologists.

## Publisher's Note

All claims expressed in this article are solely those of the authors and do not necessarily represent those of their affiliated organizations, or those of the publisher, the editors and the reviewers. Any product that may be evaluated in this article, or claim that may be made by its manufacturer, is not guaranteed or endorsed by the publisher.
