# Supplementary material for: Perceptions of Environmental Influence and Environmental Information-Seeking Behavior Among People With Asthma and COPD
Source: Front Digit Health. 2022 May 3;4:748400. doi: 10.3389/fdgth.2022.748400 (PMC9113516; doi:10.3389/fdgth.2022.748400)
Supplement: Supplementary file 2 [file Data_Sheet_1.docx]

## **Supplemental Material 1**

**Asthma survey**

***Part 1: Environmental conditions***

1) How much do you think environmental factors like air pollution, weather, pollen and climate change make your asthma symptoms worse? (matrix style question)

Responses: A great deal, a lot, a moderate amount, a little, not at all, not applicable

List

- Air pollution
- Weather
- Pollen
- Climate change/extreme weather (example: severe storms and hot temperatures)

2) Which of the **specific** environmental factors issues below do you think make your asthma symptoms worse?

Responses: Yes it makes my symptoms worse/No it doesn’t make my symptoms worse/I don’t know what this is

List

- Air Quality Index
- Particulate matter (PM 2.5)
- Particulate matter (PM 10)
- Ozone (O3)
- Nitrogen Dioxide (NO2)
- Pollen (examples: tree or weed pollen)
- Pet dander
- Mold (indoor or outdoor)
- Smoking
- Exposure to second-hand smoke

3) How much do you agree with the following statement: There are many things I can do to reduce or limit the impact of air pollution, weather, or pollen on my asthma symptoms

- Strongly agree→ strongly disagree, not applicable

4) How do you find out information about daily changes in **air pollution**? Please select ALL that apply

- - Look outside at the sky or smell the air
  - TV reports
  - local radio
  - newspaper
  - app on my phone
  - Website (example: AirNow)
  - Propeller Asthma forecast in the app
  - social media (Facebook, Twitter etc)
  - air quality flags in front of public buildings
  - I don’t look for any information about air pollution
  - I don’t know where to look for information about air pollution
  - Other
    - Please describe

5) How do you find out information about daily changes in **pollen**? Please select ALL that apply

- - Look outside
  - TV reports
  - Local radio
  - Newspaper
  - App on my phone
  - Website like (example: pollen.com)
  - Social media (Facebook, Twitter etc)
  - I don’t look for any information about pollen
  - I don’t know where to look for information about pollen
  - Other
    - Please describe

7) What do you like or not like about the sources that you use to find out information about daily changes in air pollution or pollen? Please **list** the source and **describe** for each of the options you chose in question 4

[open-ended]

***Part 2: Using Propeller***

8) How often do you look at or use the following features of the app?


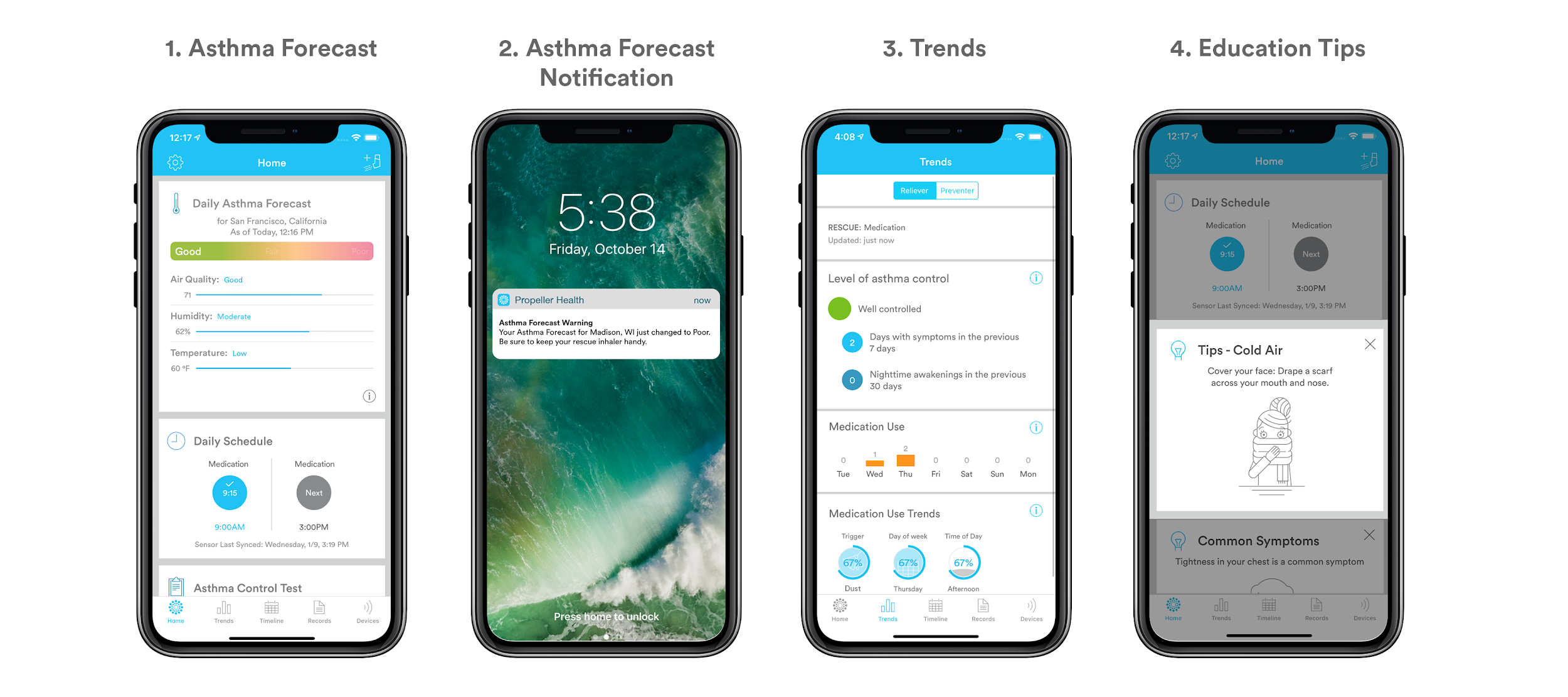


Responses: one or more times a day, one or more times a week, one or more times a month, every few months, seasonally, never, not applicable

List

- Asthma Forecast card in app
- Asthma Forecast push notification
- Tracking /Trends in app related to your triggers
- Education cards in app

9) How helpful are the following features in the app for your asthma management?

Responses: Extremely helpful → not at all helpful, not applicable

List

- Asthma Forecast card in app
- Asthma Forecast push notification
- Trends in app related to your triggers
- Education cards in app

10) Please share **why** these features are helpful or not helpful. In particular please **describe** how you use these features in your day-to-day life

[open-ended]

11) How easy is the Propeller Asthma Forecast to understand?

- Extremely easy→ not at all easy, not applicable
  - [follow up to those who select somewhat to not at all easy] Please describe why it is not easy to understand

12) How accurate do you feel the Propeller Asthma Forecast is?

- Extremely accurate→ not at all accurate, not applicable

13) On days when you see that the Propeller Asthma Forecast is poor, which of the following actions might you take? Please check ALL that apply

- - Bringing rescue med with me when go out
  - Take my daily controller medication that day
  - Adjust how I exercise (don’t exercise outside / choose to exercise inside)
  - Stay inside and avoid going outside as much as possible
  - Shut windows/doors
  - Turn on the AC/heat
  - Try to avoid my known outdoor triggers
  - Try to avoid being next to a busy road
  - Wear a face mask
  - Talk with my family and friends to let them know about the asthma forecast
  - Bring it up with my doctor
  - I don’t do anything differently
  - Not applicable, I haven’t seen a poor Asthma Forecast yet
  - Not applicable, I don’t check the Asthma Forecast
  - Other

14) As a result of the Propeller data, have you ever talked with your doctor or a clinician provider about the impact air quality, weather, or allergens have on your asthma symptoms?

- - Yes, no, I don’t see a doctor right now

15) Would you like your doctor to give you information about air quality, weather, or allergens?

- - Yes, no, I’m not sure

16) Since you started using Propeller, have you learned what specific environmental conditions (for example air pollution or weather) cause your asthma flare ups?

- - Yes, no, I don’t know, I already knew, not applicable

17) Since you started using Propeller, how confident do you feel that you can avoid having a bad flare up due to environmental conditions (for example air pollution or weather)?

- - Extremely confident → not at all confident
  - Not applicable, I don’t have flare ups due to environmental conditions

18) Since you started using Propeller, do you feel like you have had fewer symptoms because you know more about environmental conditions (for example air pollution or weather)?

- - Yes, no, I’m not sure, not applicable

19) We have plans to make improvements to the asthma forecast. How interested would you be in the following improvements?

Responses: Extremely interested→ not at all interested

List

- Asthma forecast for days in the future (1 week into the future)
- Ability to see asthma forecast for other regions (my city, my state, nationwide)
- Learning if other people in your region are having trouble with their asthma/COPD as a result of similar environmental conditions

20) In what ways could we improve the Propeller Asthma Forecast or the other ways we share information about air quality, weather, or allergens? Please describe

[open ended]

21) Which of the following topics are you interested in learning more about within the app? Please select all that apply

- - Air pollution
  - Pollen
  - Weather
  - Climate change/extreme weather (example: severe storms and hot temperatures)

21) How likely are you to recommend Propeller to a friend, family member or colleague? (scale of 1-10)

22) What is the single biggest reason for your score above? (open ended)

23) (OPTIONAL) Interested in sharing more about your thoughts or ideas about your experience using Propeller? Please write your phone number below and if selected we will reach out to set up a time for you to participate in a brief paid interview. We really value hearing your thoughts!

23) (OPTIONAL)

- What is your gender?
  - Male
  - Female
  - Prefer not to say
  - Other
- What is your race?
  - White or Caucasian
  - Black or African American
  - Hispanic or Latino
  - Asian or Asian American
  - American Indian or Alaska Native
  - Other
    - Please describe
- What is the highest grade or year of school you completed?
  - Never attended school or only attended kindergarten
  - Grades 1 through 8 (Elementary)
  - Grades 9 through 11 (Some high school)
  - Grade 12 or GED (High school graduate)
  - College 1 year to 3 years (Some college or technical school)
  - College 4 years or more (College graduate)
  - Other ___
  - Don’t know / Not sure
  - NA
- What is your annual household income from all sources?
  - Under $15,000
  - Between $15,000 and $29,999
  - Between $30,000 and $49,999
  - Between $50,000 and $74,999
  - Between $75,000 and $99,999
  - Between $100,000 and $150,000
  - Over $150,000
  - Prefer not to say
- What type of doctor do you see for asthma? Check all that apply
  - Pulmonologist
  - Allergist
  - PCP
  - I don’t see a doctor right now
  - Other

**COPD Survey**

***Part 1: Environmental conditions***

1) How much do you think environmental factors like air pollution, weather, pollen and climate change make your COPD symptoms worse? (matrix style question)

Responses: A great deal, a lot, a moderate amount, a little, not at all, not applicable

List

- Air pollution
- Weather
- Pollen
- Climate change/extreme weather (example: severe storms and hot temperatures)

2) Which of the **specific** environmental factors issues below do you think make your COPD symptoms worse?

Responses: Yes it makes my symptoms worse/No it doesn’t make my symptoms worse/ I don’t know what this is

List

- Air Quality Index
- Particulate matter (PM 2.5)
- Particulate matter (PM 10)
- Ozone (O3)
- Nitrogen Dioxide (NO2)
- Pollen (examples: tree or weed pollen)
- Pet dander
- Mold (indoor or outdoor)
- Smoking
- Exposure to second-hand smoke

3) How much do you agree with the following statement: There are many things I can do to reduce or limit the impact of air pollution, weather, or pollen on my COPD symptoms

- Strongly agree→ strongly disagree, not applicable

4) How do you find out information about daily changes in **air pollution**? Please select ALL that apply

- - Look outside at the sky or smell the air
  - TV reports
  - local radio
  - newspaper
  - app on my phone
  - Website (example: AirNow)
  - social media (Facebook, Twitter etc)
  - Air quality flags in front of public buildings
  - I don’t look for any information about air pollution
  - I don’t know where to look for information about air pollution
  - Other
    - Please describe

5) How do you find out information about daily changes in **pollen**? Please select ALL that apply

- - Look outside
  - TV reports
  - Local radio
  - Newspaper
  - App on my phone
  - Website like (example: pollen.com)
  - Social media (Facebook, Twitter etc)
  - I don’t look for any information about pollen
  - I don’t know where to look for information about pollen
  - Other
    - Please describe

4) What do you like or not like about the sources that you use to find out information about daily changes in air pollution or pollen? Please describe for each of the options you chose in the previous question

[open-ended]

***Part 2: Using Propeller***

6) How often do you look at or use the following features of the app?


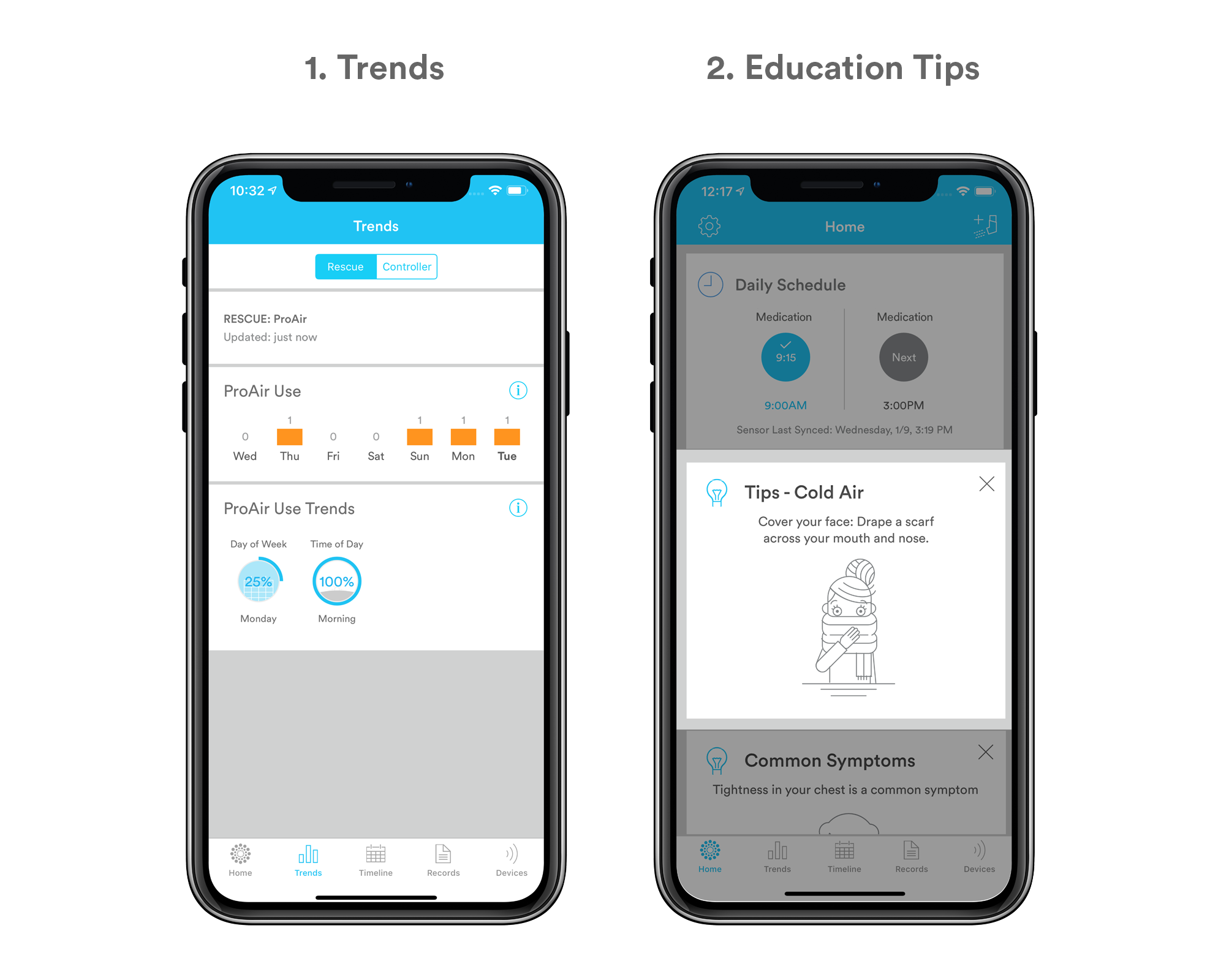


Responses: one or more times a day, one or more times a week, one or more times a month, every few months, seasonally, never, not applicable

List

- Tracking /Trends in app related to your triggers
- Education cards in app

7) How helpful are the following features in the app for your COPD management?

Responses: Extremely→ not at all helpful, not applicable

List

- Trends in app related to your triggers
- Education cards in app

8) Please explain why these features are helpful or not helpful. In particular please describe how you use these features in your day-to-day life

[open-ended]

9) As a result of the Propeller data, have you ever talked with your doctor about the impact air quality, weather, or allergens have on your COPD symptoms?

- - Yes, no, I don’t see a doctor right now

10) Would you like your doctor to give you information about air quality, weather, or allergens?

- - Yes, no, I’m not sure

11) Since you started using Propeller, have you learned what specific environmental conditions (for example air pollution or weather) cause your COPD flare ups?

- - Yes, No, I don’t know, I already knew, not applicable

12) Since you started using Propeller, how confident do you feel that you can avoid having a bad flare up due to environmental conditions (for example air quality or weather)?

- - Extremely → not at all confident, not applicable

13) Since you started using Propeller, do you feel like you have had fewer symptoms because you know more about environmental conditions (air quality or weather)?

- - Yes, no, I’m not sure, not applicable

14) We have plans to add new features to help people learn about air quality and weather. How interested would you be in the following potential features?

Responses: Extremely interested→ not at all interested

List

- A COPD forecast that provides personalized information about how your local environmental conditions could impact their COPD that day. The forecast provides 3 possible ratings: “Good”, “Fair”, or “Poor,” which indicates how likely the conditions are to cause a flare-up for you
- Learning if other people in your region are having trouble with their COPD as a result of similar environmental conditions

15) In what ways could we improve the ways we share information about air quality, weather, or allergens? Please describe

[open ended]

16) Which of the following topics are you interested in learning more about within the app?

- - Air pollution
  - Pollen
  - Weather
  - Climate change/extreme weather (example: severe storms and hot temperatures)

17) How likely are you to recommend Propeller to a friend, family member or colleague? (scale of 1-10)

18) What is the single biggest reason for your score above? (open text field)

19) (OPTIONAL) Interested in sharing more about your thoughts or ideas about your experience using Propeller? Please write your phone number below and if selected we will reach out to set up a time for you to participate in a brief paid interview. We really value hearing your thoughts!

(OPTIONAL)

- What is your gender?
  - Male
  - Female
  - Prefer not to say
  - Other
- What is your race?
  - White or Caucasian
  - Black or African American
  - Hispanic or Latino
  - Asian or Asian American
  - American Indian or Alaska Native
  - Other
    - Please describe
- What is the highest grade or year of school you completed?
  - Never attended school or only attended kindergarten
  - Grades 1 through 8 (Elementary)
  - Grades 9 through 11 (Some high school)
  - Grade 12 or GED (High school graduate)
  - College 1 year to 3 years (Some college or technical school)
  - College 4 years or more (College graduate)
  - Other ___
  - Don’t know / Not sure
  - NA
- What is your annual household income from all sources?
  - Under $15,000
  - Between $15,000 and $29,999
  - Between $30,000 and $49,999
  - Between $50,000 and $74,999
  - Between $75,000 and $99,999
  - Between $100,000 and $150,000
  - Over $150,000
  - Prefer not to say
- What type of doctor do you see for COPD? Check all that apply
  - Pulmonologist
  - Allergist
  - PCP
  - I don’t see a doctor right now
  - Other

**Specific survey questions of interest**

| **Theme** | **Question** | **Response** |
| --- | --- | --- |
| **Preferred sources for environmental information** | How do you find out information about daily changes in air pollution or pollen? (Please select ALL that apply) | - Look outside - Talk with my doctor - Talk with a friend or family member - Check the TV reports - Listen to local radio - Check a newspaper - Check on an app on my phone - Check a website - Check Propeller Asthma forecast in the app - Check social media (Facebook, Twitter etc) - Look at air quality flags in front of public buildings - I don’t look for any information about air pollution or pollen - I don’t know where to look for information about air pollution or pollen - Other |
| **Knowledge and perceptions of environmental influence** | Which of the specific environmental factors do you think make your asthma/COPD symptoms worse? (Yes it makes my symptoms worse/No it does not make my symptoms worse/I’m not sure) | - Air Quality Index - Particulate matter (PM 2.5) - Particulate matter (PM 10) - Ozone (O3) - Nitrogen Dioxide (NO2) - Pollen - Pet dander - Mold - Tobacco smoke |
| **Sense of agency to practice self-management** | How much do you agree with the following statement: There are many things I can do to reduce or limit the impact of air pollution, weather, or pollen on my asthma/COPD symptoms | - Strongly agree - Agree - Neither agree nor disagree - Disagree - Strongly disagree - Not applicable |
|  | On days when you see that the Propeller Asthma Forecast is poor, which of the following actions might you take? (Please select ALL that apply) | - Bring my rescue medication with me when go out - Take my daily controller medication that day - Adjust how I exercise (don’t exercise outside/choose to exercise inside) - Stay inside and avoid going outside as much as possible - Shut windows/doors - Turn on the AC/heat - Try to avoid my known outdoor triggers - Try to avoid being next to a busy road - Wear a face mask - Talk with my family and friends to let them know about the asthma forecast - Bring it up with my doctor - I don’t do anything differently - Not applicable, I haven’t seen a poor Asthma Forecast yet - Not applicable, I don’t check the Asthma Forecast - Other |

**Table S2.** Specific survey questions of interest.
